# Experiential Measures Can Be Used as a Proxy for Language Dominance in Bilingual Language Acquisition Research

**DOI:** 10.3389/fpsyg.2018.01809

**Published:** 2018-10-17

**Authors:** Sharon Unsworth, Vicky Chondrogianni, Barbora Skarabela

**Affiliations:** ^1^Centre for Language Studies, Radboud University, Nijmegen, Netherlands; ^2^School of Philosophy, Psychology and Language Sciences, The University of Edinburgh, Edinburgh, United Kingdom

**Keywords:** language dominance, language exposure, language use, bilingual children, relative proficiency

## Abstract

Language dominance is a multidimensional construct comprising several distinct yet interrelated components, including language proficiency, exposure and use. The exact relation between these components remains unclear. Several studies have observed a (non-linear) relationship between bilingual children’s amount of exposure and absolute proficiency in each language, but our understanding of the relationship between language exposure and use and relative proficiency is limited. To address this question, we examined whether experiential-based measures of language dominance, operationalised here in the narrow sense of relative language proficiency, can provide an efficient alternative to the more labor-intensive performance-based measures often used in the literature. In earlier work, Unsworth (a) examined the relationship between relative proficiency and language exposure and use in a group of English–Dutch bilingual preschool children residing in the Netherlands. This study expands these findings by examining Dutch–English preschool children of the same age residing in the United Kingdom in order to cover the full dominance continuum. Participants were 35 simultaneous bilingual children (2;0–5;0) exposed to English and Dutch, 20 resident in the Netherlands and 15 in the United Kingdom. Relative amount of language exposure and use were estimated using a parental questionnaire. To obtain performance-based measures of language proficiency, children’s spontaneous speech was recorded during a half-hour play session in each language. The transcribed data were used to derive MLU (words), average length of the longest five utterances, the number of different verb and noun types. Single word vocabulary comprehension was assessed using standardized tests in both languages. Following Yip and Matthews (2006), relative proficiency was operationalised using differentials. In line with Unsworth (2016a), English-dominant children typically had less than approx. 35% exposure to Dutch and used Dutch less than approximately 30% of the time. Curve-fitting analyses revealed that non-linear models best fit the data. Logistic regression analyses showed that both exposure and use were good predictors of dominance group membership assigned using the same approach as Unsworth (2016a), that is, using SDs. Dominance groups derived independently using cluster analyses overlapped with the groups derived using SDs, confirming that relative amount of exposure and use can be used as a proxy for language dominance.

## Introduction

Bilingual children’s language development is affected by certain characteristics of their language learning experience. For example, numerous studies have found that bilingual children’s rate of acquisition in vocabulary and grammar is often predicted by the relative amount of input to which they are exposed in their two languages (e.g., Hoff et al., 2012; see Unsworth, 2016b for a recent overview). Similarly, a number of recent studies have demonstrated that children’s own language use has a significant impact on their language development across a range of domains (e.g., Bohman et al., 2010; Ribot et al., 2017). Almost all of these studies focus on the relationship between (relative) measures of language experience and children’s *absolute* proficiency in one or both of their two languages. Considerably fewer studies (e.g., Bedore et al., 2012) have explored the relationship between these experiential variables and children’s *relative* proficiency, that is, how well children perform in one language compared with the other.

Bilinguals who are equally proficient – or balanced – in their two languages are rare (e.g., Grosjean, 1982, 2010). Whilst there is a broad consensus that bilingual children, even if exposed to both languages from birth, are more proficient or *dominant* in one of their two languages, how best to define language dominance remains a contentious issue. Part of the challenge in defining language dominance is that it involves a multidimensional construct consisting of several distinct yet interrelated components, including language use, language input, and language proficiency (see Montrul, 2016 and Silva-Corvalán and Treffers-Daller, 2016 for relevant discussion). In the present study, we adopt a narrow definition of *language dominance*, focusing on relative proficiency across the two languages, in order to explore the relationship between these various component parts.

In the bilingual acquisition literature, dominance is typically operationalised using either *performance-based measures* such as mean length of utterance (MLU) or lexical diversity (e.g., Cantone et al., 2008) or *experiential-based measures* such as amount of exposure or country of residence (e.g., Döpke, 1992; Argyri and Sorace, 2007; Foroodi-Nejad and Paradis, 2009; Serratrice et al., 2009; Tuller et al., 2018). Whilst the former are arguably more objective, they are considerably more time-consuming and consequently more expensive than the latter; experiential-based measures are certainly cheaper and quicker to administer but they are often considered subjective and rather crude in comparison with performance-based measures. It is, however, unclear whether this is indeed the case.

The goal of the present study is, therefore, to assess whether and to what extent experiential-based measures can be used as a proxy for language dominance in bilingual language development research. The paper is organized as follows. First, we briefly summarize the main findings concerning the relationship between language exposure and use and *absolute* language proficiency. Next, we review the more limited previous literature examining the relationship between these two experiential variables and *relative* language proficiency, including a study by Unsworth (2016a) on English–Dutch bilingual preschoolers in the Netherlands, which serves as the starting point for the present study. Subsequently, we combine data from this earlier study with new data from English–Dutch bilingual preschoolers in the United Kingdom in order to explore the relationship between language exposure and use and language dominance across the whole dominance continuum. Our main finding is that in line with Bedore et al. (2012) and Unsworth (2016a), there is a moderate to strong non-linear relationship between language exposure and use, on the one hand, and relative language proficiency, on the other, suggesting that experiential-based measures can indeed be used as a proxy for performance-based measures of language dominance.

## *Absolute* and *Relative* Proficiency

### Language Exposure and Use and *Absolute* Proficiency

Bilingual children’s language experience varies considerably. Whilst some children hear the minority language from both parents, others receive minority language input from one parent only. For some children, their parent(s) are the only source of the minority language, whereas others have access to minority language input from other family members and friends. Furthermore, some children hear language input from native speakers only, yet others also hear language input – sometimes exclusively so – from non-native speakers. There is also a difference in the availability of TV, apps and other media across different languages. Taken together, this variability in how bilingual children experience their two languages means that there is considerable variation between and sometimes also within children in terms of the quantity and quality of language exposure and use. This variation has been observed to predict bilingual children’s developing language skills across a range of linguistic domains, language combinations and sociolinguistic settings.

Input quantity effects have been observed for a range of different domains of bilingual children’s language proficiency, such as vocabulary (Gathercole and Thomas, 2009; Thordardottir, 2011), aspects of morphosyntax such as MLU (Place and Hoff, 2011; Hoff et al., 2012) and verbal morphology (Nicoladis et al., 2007; Blom, 2010; Paradis et al., 2011), as well as certain phonological abilities (Sundara et al., 2008; Nicoladis and Paradis, 2011). The relationship between relative amount of exposure and language skills has been found to be non-linear in nature. This means, for example, that once bilingual children reach a certain input threshold, they score on a par with monolingual peers but beyond that threshold, the relationship between exposure and proficiency is more limited, if present at all (e.g., Pearson et al., 1997; Cattani et al., 2014; Thordardottir, 2015). Differential effects of input have been observed for toddlers (e.g., Place and Hoff, 2011), preschoolers (e.g., Paradis et al., 2011) and primary school children (e.g., Gathercole and Thomas, 2009), and in both simultaneous (e.g., Unsworth, 2013) and successive (e.g., Chondrogianni and Marinis, 2011; Paradis, 2011) bilingual children, and in minority language (e.g., Hoff et al., 2012) and bilingual (e.g., Gathercole and Thomas, 2009) sociolinguistic contexts. In short, there is considerable evidence for a robust relationship between amount of language exposure and rate of acquisition in bilingual language development. Most of the aforementioned studies concerned bilingual children who were still relatively young and therefore unlikely to have reached their end state in one or both of their two languages. In other words, most of the literature on input effects deals with *rate* of acquisition rather than the end state. It remains unclear whether amount of language exposure in early childhood is also a strong predictor of children’s long-term outcomes (but see e.g., Montrul, 2008 for evidence that it likely is).

In addition to language exposure, children’s own language use has also been found to play a significant role in their bilingual development. Several recent studies have shown that the extent to which children actively speak the language in question significantly predicts their developing language abilities. For example, a study on the early semantic and morphosyntactic development of Spanish–English bilinguals found that children’s language use was a significant predictor of both domains in both languages, whereas input was only relevant for both domains in English (Bohman et al., 2010). Similar findings were reported for children’s morphosyntactic development and vocabulary size (e.g., Montrul, 2008; Paradis, 2011; Hammer et al., 2012). More recently, Ribot et al. (2017) also observed that after controlling for input effects, language use at 30 months predicted bilingual Spanish–English children’s expressive vocabulary skills at 36 and 42 months, but this was not the case for receptive skills. More specifically, children whose use in English was greater than their input in English (i.e., children who sometimes switched to English when spoken to in Spanish) had higher expressive vocabulary scores and their scores increased at a faster rate than children whose output in English was less than their input in English (i.e., children who sometimes switched to Spanish when spoken to in English). The effect of language use on children’s Spanish skills was not assessed. To summarize, there is emerging evidence suggesting that in addition to language exposure, language use has also been found to predict unique variance in bilingual children’s absolute proficiency in one or both of their languages.

### Language Exposure and Use and *Relative* Proficiency

As noted above, there are comparatively fewer studies examining the relationship between language exposure and use and *relative* rather than *absolute* language proficiency, even though relative language exposure is often used as a proxy for language dominance (e.g., Foroodi-Nejad and Paradis, 2009). It has been argued that using relative measures of exposure to predict absolute measures of language skill fails to capture variation in the overall amount of child-directed speech to which children are exposed (i.e., their absolute exposure), and that this variability can be controlled for by comparing relative measures of exposure with relative measures of proficiency, particularly when the goal is to better understand patterns of language balance or dominance (Grüter et al., 2014), as is the case here.

There are a number of ways in which absolute language proficiency scores in two languages can be combined to provide some measure of *relative* language proficiency or language dominance (in the narrow sense in which it is used here). One common approach in the bilingual language acquisition literature, which is also adopted here, is to use differentials (e.g., Yip and Matthews, 2006). Differentials involve subtracting a child’s score on one language from his or her score on the other language; this method can in principle be adopted with any measure of language proficiency, although scores need to be standardized before subtraction if they are to be directly comparable across different measures (for relevant discussion see Birdsong, 2016 and Treffers-Daller and Korybski, 2016).

One of the few studies using exposure and use to predict relative proficiency is Bedore et al. (2012). A large sample of 5-year-old Spanish-English bilinguals (*n* = 1029) participated in the semantics and morphosyntax subtests of the BESOS (Bilingual English Spanish Oral Language Screening, developed by the same authors), both productive tasks. Following the standard practice in the field, measures of language experience were estimated from parental questionnaires. Children were divided into dominance groups (i.e., functionally monolingual in Spanish, bilingual Spanish dominant, balanced bilingual, bilingual English dominant, functionally monolingual in English) based on experiential-based measures (i.e., differences in their relative language exposure and use in English and Spanish) and on performance-based measures (i.e., differences in their scores on the morphosyntactic and semantics subtests in English and Spanish). The authors observed that the dominance profiles derived from the two experiential-based measures were more consistent with each other than those derived from the two performance-based measures, although this is perhaps unsurprising given that language exposure and use were so highly correlated in their sample (*r* = 0.95). A combined current usage score based on these two factors was found to account for more variance in children’s relative morphosyntactic and semantic proficiency than age of first exposure. The authors concluded that current usage should therefore be included in assessing dominance patterns in (5-year-old) bilingual children. As the authors note, however, given that language use has been found to relate to language development differently from language input (Bohman et al., 2010), it is nevertheless important to consider the two separately.

In a smaller scale study with younger children, Unsworth (2016a) investigated the relationship between relative proficiency and language exposure, on the one hand, and language use, on the other. More specifically, Unsworth compared a number of performance-based commonly used measures of language dominance derived from spontaneous speech samples (i.e., MLU and various measures of lexical diversity) with experiential-based measures derived from parental questionnaires to explore whether the latter could reasonably be used as a proxy for the former. Participants were 18 simultaneous bilingual English-Dutch children aged between 2 and 4 years old. Despite being strongly correlated with each other (*r* = 0.80), the children’s patterns of language exposure and use related differently to their relative proficiency, as determined by differentials (following Yip and Matthews, 2006). More specifically, whereas the children classified as Dutch-dominant on the basis of their differential scores all had at least 65% relative exposure to Dutch, they used Dutch almost exclusively, at least 90% of the time. On the basis of these findings, Unsworth concluded that, in line with previous work on the relationship between exposure and absolute proficiency (see above), experiential-based measures may be used as a proxy for language dominance.

The children in Unsworth’s study were all resident in the Netherlands and were all found to be Dutch-dominant or balanced. To fully understand the potential of using amount of exposure as proxy for language dominance, it is important to include children from the whole dominance continuum. For this reason, the present study combines the original data in Unsworth (2016a) with new data from English–Dutch bilingual children resident in the United Kingdom to create a larger sample, including bilingual children in a primarily English-speaking environment, and allowing us to conduct a range of analyses. Our research questions are as follows:

(1) To what extent do experiential-based factors such as relative language exposure and use predict bilingual children’s relative language proficiency?
(2) Can language exposure and use reliably classify bilingual children into language dominance groups?
(3) Could experiential-based measures be used as a proxy for language dominance?

Based on previous work focusing on absolute proficiency, as well as Bedore et al. (2012) and Unsworth (2016a), we predict that relative proficiency scores will correlate strongly with both measures of language exposure and use. The exact nature of this relationship, however, will not necessarily be the same (e.g., Ribot et al., 2017) and it will not be linear (e.g., Thordardottir, 2011; Cattani et al., 2014). More specifically, assuming the United Kingdom-based children will pattern similarly to their peers in the Netherlands, we expect in answer to the second research question that children classified as English-dominant should have no more than 35% exposure to Dutch and no more than 10% of their language output in Dutch. Similarly, children classified as balanced bilinguals on the basis of their differential scores should hear and use Dutch more than the English-dominant children, but less than the values observed for Dutch-dominant children in the original study (i.e., below 65% for language exposure and 90% for use). If these expectations are borne out, then experiential-based measures of language exposure and use could arguably be used as a proxy for language dominance.

## Materials and Methods

### Participants

Participants were 35 simultaneous bilingual children exposed to English and Dutch, 20 resident in the Netherlands (age range: 2;9 – 4;6; *M* = 3;9; *SD* = 0;7; 7 girls, 18 taken from Unsworth, 2016a plus two additional children) and 15 resident in the United Kingdom (age range: 2;0 – 5;1; *M* = 3;5; *SD* = 1;1; 10 girls). All but two children in the Netherlands and all but one child in the United Kingdom were exposed to both languages from birth; the three exceptions were all exposed to both languages before the age of two. Their inclusion in the analyses did not affect any of the results.

The children in the Netherlands were almost all being raised following the *one parent, one language* approach: in twelve families the mother mostly or always spoke English to the child and the father mostly or always spoke Dutch, and in seven families this pattern was reversed. In the remaining family the mother spoke slightly more Dutch than English and the father always spoke Dutch. All but one child had siblings: eleven children were first-born, eight had one older sibling and one was the youngest of three. With four exceptions, all (older or same-age siblings) almost always spoke Dutch with the participating child. There were 12 children attending daycare, seven attending school and one child transitioning from daycare to school; the language of communication at all schools and daycares was Dutch. All participating families were high SES: with the exception of one father who had completed secondary education only, both parents had a university degree.

The United Kingdom sample was more heterogeneous and included families using the *one parent, one language* approach and families where both parents spoke the same language. More specifically, in five families the mother mostly spoke Dutch to the child and the father always spoke English, in one family this pattern was reversed, and in seven families both parents spoke Dutch to the child. In the remaining two families, both parents mostly (or always) spoke English. All but four children had siblings: four children were first born, six were the youngest with one or two older siblings, one was the middle child of three. The main language of communication amongst siblings was English. Thirteen children were exposed to English at nursery/preschool; one child stayed at home with an English-speaking childminder; and one child stayed at home with her English-speaking father. All participating families were high SES: with the exception of one father who had completed further education college, all parents held a university degree.

### Method and Procedure

To examine performance-based and experience-based measures of language dominance in bilingual English–Dutch, we used three sources of data: (1) children’s spontaneous speech productions in naturalistic interactions with a parent or researcher in each language; (2) children’s receptive vocabulary skills; and (3) parental questionnaire data.

#### Spontaneous Speech Recordings

All children were video-recorded in a half-hour session in each language. In the Netherlands, each child was recorded interacting with the parent who normally used the language in question with the child. The parent was asked to interact with their child as they would usually do. Due to the heterogeneity of the United Kingdom sample and the unavailability of some of the English-speaking parents, children were usually recorded interacting with their parents (primarily mothers) in Dutch, whereas for English, all but one child were recorded interacting with a (near-)native-speaker research assistant. All children in the Netherlands and all but two children in the United Kingdom were recorded in their homes. One was recorded at nursery rather than home because the child would only speak English at nursery; the other was recorded in the university’s developmental lab at the parent’s request. Irrespective of location or interlocutor, all children participated in similar activities in both languages, typically involving playing with puzzles or lego, looking at picture books or drawing.

The data were transcribed in CLAN/CHAT (MacWhinney, 2000) by a (near-)native-speaker of English or Dutch and checked for accuracy by another assistant. The following were excluded from analysis: incomplete utterances (e.g., trailing off), direct imitations of interlocutor, self-repetition, series of utterances (e.g., counting), utterances containing unintelligible parts, as well as any utterances in or containing words from the other language (with the exception of proper names and accepted loanwords).

In both samples and languages, we calculated the MLU in words as well as the average length of the longest five utterances in the sample (Upper Bound, UB5). The FREQ function was used to generate a list of words for each sample and the number of different verbs (VERBS) and nouns (NOUNS) was extracted and counted manually; any ambiguities were checked against the original transcript. Given the differences in sample size across children and languages and following common procedure in the field, data were analyzed for the first 100 utterances only; where fewer than 100 utterances were available, all utterances were included. For the children in the Netherlands, all but one produced at least 100 utterances in 30 min in Dutch. For English, 11 children did not reach 100 utterances. For the children in the United Kingdom, all produced 100 utterances in Dutch but four produced fewer than 100 utterances in English.

#### Receptive Vocabulary Skills

In addition to the indicators of language abilities from children’s spontaneous speech, we also assessed their receptive vocabulary skills, using standardized vocabulary tests. The PPVT-III-NL was used for Dutch (Dunn et al., 2005). For English, children in the Netherlands were given the PPVT-4 (Dunn and Dunn, 2007) or BPVS-2 for English, depending on the variety of English the child was exposed to; children in the United Kingdom completed the BPVS III (Dunn et al., 1997). Raw scores were converted to standard scores following the procedure in the manual; a score of between 85 and 115 indicates age-appropriate development for a monolingual child. The analyses rely on raw scores as standard scores were not available for children under the age of three. We used the raw scores to compare performance within and across children and languages, and to provide a general assessment of children’s lexical knowledge.

#### Parental Questionnaire

Information concerning the children’s language experience was collected using an extensive parental questionnaire, the BiLEC (Bilingual Language Experience Calculator; Unsworth, 2013, following Gutiérrez-Clellen and Kreiter, 2003; Paradis, 2011). Parents were asked to indicate where and with whom the child spent time on an average day in the week and an average day at the weekend, for how long, and which language(s) each person used when addressing the child, as well as time spent on extra-curricular activities and the language(s) in which these occurred. This information was used to calculate proportion of language exposure to Dutch vs. English at the current time (see Unsworth, 2013 for more details). Comparable information was gathered concerning the child’s output with the same interlocutors and this was used to calculate current proportion of language use in Dutch vs. English at the current time. Finally, parents were asked about children’s patterns of language exposure in the past and this was used to calculate their *cumulative* length of exposure (see Unsworth, 2013 for more details).

#### Procedure

Children were tested on separate occasions in each language, with no more than 2 weeks between sessions, and for almost all children, the following test order was used: vocabulary task followed by spontaneous speech production. At the end of one of the two sessions, the parent completed the background and language experience questionnaire during a short informal interview.

## Results

The experiential-based measures derived from the parental questionnaire are presented in **Table 1**. At the group level, children had a relatively balanced exposure to their two languages, but they used Dutch more frequently than English in both locations. There was considerable individual variation, suggesting a range of patterns of language exposure and use in the dataset.

**Table 1:** Mean age, relative language exposure and use in Dutch, and cumulative length of exposure to Dutch and English (*N* = 35).

	Age (months)	Average proportion of weekly input in Dutch (%)	Average proportion of weekly output in Dutch (%)	Cumulative length of exposure to Dutch (months)	Cumulative length of exposure to English (months)
Mean	43.2	53	59	24	18
*SD*	9.8	18	35	0.9	0.6


The performance-based measures derived from the spontaneous speech samples and the vocabulary tests are given in **Table 2**. As noted in the Section “*Materials and Methods*,” differential scores for vocabulary were calculated using the raw scores. The mean standard score for vocabulary was 100 (*SD* = 11.3) for English and 99.5 (*SD* = 15.2) for Dutch. At the group level, children tended to produce longer sentences in Dutch than in English, but the number of different nouns and verbs was more comparable across the two languages, as were the vocabulary scores. Once again, there was considerable variation between children, suggesting a range of patterns of language proficiency in the dataset.

**Table 2:** Mean absolute and relative proficiency scores (*N* = 35).

Variable	Absolute scores				Relative scores	
		
	Dutch		English		Differential (Dutch – English)	
			
	*M*	*SD*	*M*	*SD*	*M*	*SD*
MLU (in words)	2.99	0.92	2.44	1.00	0.54	0.99
UB5 (in words)	8.43	3.80	7.97	4.58	0.45	4.89
VERBS (#)	16.1	8.71	14.9	9.13	1.14	11.5
NOUNS (#)	17.0	8.62	18.1	9.07	-1.14	10.4
VOCAB (raw score)	50.9	18.0	46.5	19.6	4.40	15.9


Comparing children’s scores in the two languages, MLU was significantly higher in Dutch than English (*t*(34) = 3.21, *p* = 0.003) but there were no significant differences between languages on the other three scores (VERBS: *t*(34) = 0.600, *p* = 0.552; NOUNS: *t*(34) = -0.653, *p* = 0.518; UB5: *t*(34) = 0.550, *p* = 0.586; VOCAB: *t*(34) = 1.63, *p* = 0.112).

### Establishing the Strength and Shape of the Relationship Between Experience-Based and Performance-Based Measures

To explore the relationship between the experience-based and performance-based measures we first conducted bivariate correlational analyses to establish the strength of any relationships between the two sets of variables. Amount of exposure correlated significantly with differentials for MLU (*r* = 0.57, *p* = 0.001), UB5 (*r* = 0.57, *p* = 0.001), VERBS (*r* = 0.65, *p* < 0.001), NOUNS (*r* = 0.49, *p* = 0.002) and VOCAB (*r* = 0.49, *p* = 0.003) and language use correlated significantly with differentials for MLU (*r* = 0.77, *p* < 0.001), UB5 (*r* = 0.67, *p* < 0.001), VERBS (*r* = 0.84, *p* < 0.001), NOUNS (*r* = 0.55, *p* = 0.001) and VOCAB (*r* = 0.45, *p* = 0.006) (cf. original study, where there were only significant correlations with MLUdiff and VERBS_diff_).

As a next step, linear (y = b_0_ + b_1_x), quadratic (y = b_0_ + b_1_x + b_2_x^2^) and cubic (y = b_0_ + b_1_x + b_2_x^2^+ b_3_x^3^) relationships were estimated using the *Curve Estimation* function in IBM SPSS v.25. The goal of this analysis was to determine whether the relation between the experience- and performance-based measures was best accounted for by non-linear rather than linear regression models (following Thordardottir, 2011 and Bedore et al., 2012). The results are presented in **Table 3**.

**Table 3:** Summary of regression models using different estimation methods (incremental *F*-values and *R*^2^).

	Type of regression model		
	
Variable	Linear	Quadratic	Cubic
**MLU_diff_**			
Exposure	*F* = 23.9^∗∗∗^ *R*^2^ = 0.42	*F* = 13.6^∗∗∗^ *R*^2^ = 0.04	*F* = 9.7^∗∗∗^ *R*^2^ = 0.02
Use	*F* = 50.9^∗∗∗^ *R*^2^ = 0.61	*F* = 26.1^∗∗∗^ *R*^2^ = 0.01	*F* = 17.2^∗∗∗^ *R*^2^ = 0.01
**UB5_diff_**			
Exposure	*F* = 21.6^∗∗∗^ *R*^2^ = 0.40	*F* = 10.5^∗∗∗^ *R*^2^ = 0.00	*F* = 7.3^∗∗∗^ *R*^2^ = 0.02
Use	*F* = 27.0^∗∗∗^ *R*^2^ = 0.45	*F* = 14.0^∗∗∗^ *R*^2^ = 0.02	*F* = 9.4^∗∗∗^ *R*^2^ = 0.01
**VERBS_diff_**			
Exposure	*F* = 33.8^∗∗∗^ *R*^2^ = 0.51	*F* = 18.3^∗∗∗^ *R*^2^ = 0.03	*F* = 13.1^∗∗∗^ *R*^2^ = 0.03
Use	*F* = 83.3^∗∗∗^ *R*^2^ = 0.72	*F* = 42.0^∗∗∗^ *R*^2^ = 0.01	*F* = 27.6^∗∗∗^ *R*^2^ = 0.00
**NOUNS_diff_**			
Exposure	*F* = 13.3^∗∗∗^ *R*^2^ = 0.29	*F* = 6.5^∗∗^ *R*^2^ = 0.00	*F* = 6.0^∗∗^ *R*^2^ = 0.08
Use	*F* = 14.3^∗∗∗^ *R*^2^ = 0.30	*F* = 7.1^∗∗^ *R*^2^ = 0.01	*F* = 4.6^∗∗^ *R*^2^ = 0.00
**VOCAB_diff_**			
Exposure	*F* = 10.2^∗∗^ *R*^2^ = 0.24	*F* = 5.8^∗∗^ *R*^2^ = 0.03	*F* = 3.8^∗^ *R*^2^ = 0.00
Use	*F* = 8.5^∗∗^ *R*^2^ = 0.20	*F* = 4.3^∗^ *R*^2^ = 0.01	*F* = 3.1^∗^ *R*^2^ = 0.02


*^∗^*p* < 0.05, ^∗∗^*p* < 0.01, ^∗∗∗^*p* < 0.001.*

In all cases, the linear plus non-linear models accounted for more variance (i.e., had a higher total incremental *R*^2^ value) than the linear models alone, although the additional unique variance explained by the non-linear models was negligible in certain cases. The amount of variance explained for NOUNS_diff_ was lower than for the other performance-based measures of relative proficiency, especially MLU_diff_ and VERBS_diff_, and across most measures language use was a better predictor than language exposure.

### Predicting Dominance Group Membership Using Experiential Variables

**FIGURE 1:**
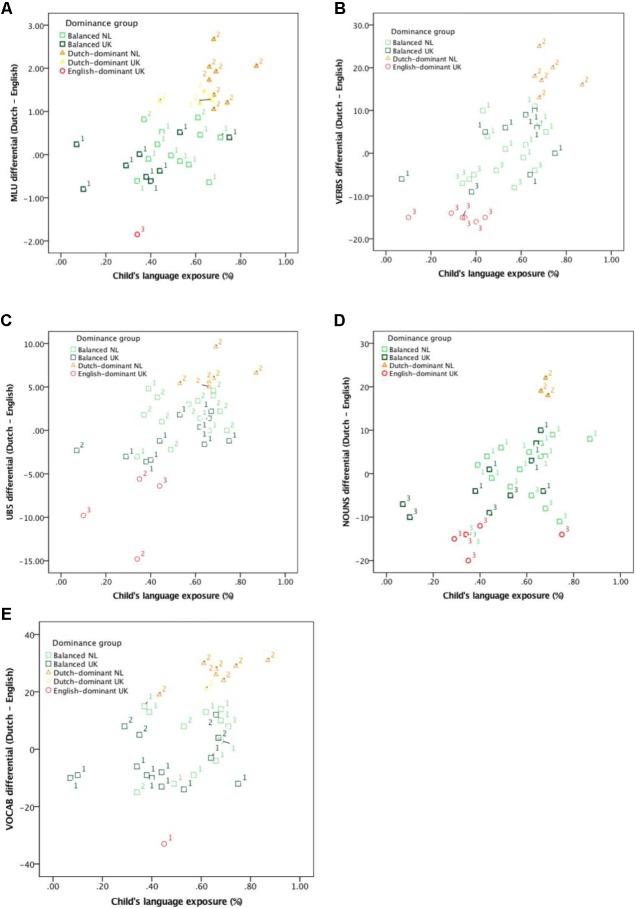
Scatterplots showing relationship between relative language exposure (% Dutch) and relative language proficiency as measured by differentials for MLU **(A)**, VERBS **(B)**, UB5 **(C)**, NOUNS **(D)**, and VOCAB **(E)** for two different classifications dominance groups based on SDs and *k*-means cluster analysis groups. 1 = Balanced, 2 = Dutch-dominant, 3 = English-dominant according to the *k*-means cluster analysis.

**FIGURE 2:**
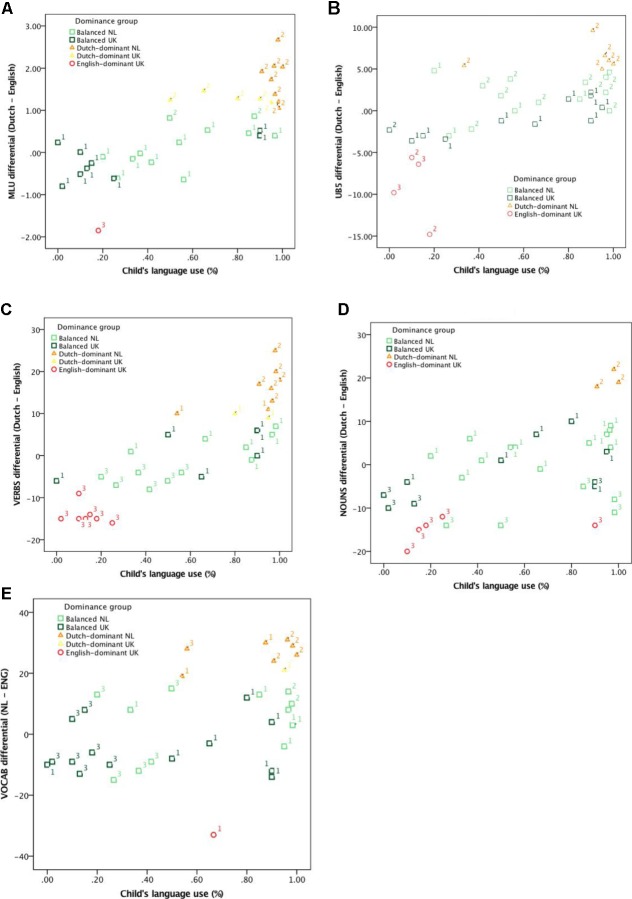
Scatterplots showing relationship between relative language use (% Dutch) and relative language proficiency as measured by differentials for MLU **(A)**, VERBS **(B)**, UB5 **(C)**, NOUNS **(D)**, and VOCAB **(E)** for two different classifications dominance groups based on SDs and *k*-means cluster analysis groups. 1 = Balanced, 2 = Dutch-dominant, 3 = English-dominant according to the *k*-means cluster analysis.

Children were classified as Dutch-dominant, balanced, and English-dominant in exactly the same way as in the original study: children were classified as dominant in one of their two languages when there was a difference of greater than 1 SD between the two, and when this difference was less than 1 SD, children were classified as balanced. For example, children whose MLU in Dutch was greater than their MLU in English by at least 0.99 words (cf. **Table 2**) were considered Dutch-dominant, children whose MLU in English was greater than their MLU in Dutch by at least 0.99 words were considered English-dominant and children with a differential score less than 0.99 words were classified as balanced. As noted in Unsworth (2016a), using a one-word difference in MLU as a measure for dominance is in line with earlier work (e.g., Bernardini and Schlyter, 2004). As in Unsworth (2016a), we extend this approach to the remaining performance-based variables using SDs as our guideline for dominance classification (see Discussion section for further consideration of this approach). The distribution of children in the three dominance groups, along with their country of residence, indicated by color in both cases, is presented in **Figure 1** in relation to language exposure and in **Figure 2** in relation to language use.

Despite being resident in the United Kingdom, only a few of the children who were added to the original dataset as part of this study were English-dominant, and when children were classified into dominance groups on the basis of MLU_diff_, there was just one.

A series of multinomial logistic regression analyses were run (in IBM SPSS v.25) to investigate how well language exposure and use predicted group membership (balanced, Dutch-dominant, English-dominant) for the dominance groups derived on the basis of the four different performance-based measures (MLU_diff_, VERBS_diff_, NOUNS_diff_ and UB5_diff_). The reference category was set to English-dominant. The extent to which language use and exposure correctly predicted group membership, the overall success rate of the model in doing so, as well as an estimation of the amount of variance explained (Nagelkerke’s *R*) are given in **Table 4**. Values between 80 and 89% are considered fair, while rates above 90% are good (Plante and Vance, 1994).

**Table 4:** Estimates of language exposure and use on dominance group membership based on differential scores (% and number of children correctly predicted).

	Prediction success				Variance explained	
		
	Overall	NL-dom	Balanced	ENG-dom	Nagelkerke’s R (standard regression model)	*R*^2^ (Firth penalized regression model)
**MLU_diff_**						
Exposure	83%	92%(12/13)	81% (17/21)	0% (0/1)	0.50^∗∗∗^	0.32^∗∗^
Use	77%	85% (11/13)	76% (16/21)	0% (0/1)	0.55^∗∗∗^	0.35^∗∗∗^
**UB5_diff_**						
Exposure	74%	17% (1/6)	96% (24/25)	25% (1/4)	0.37^∗∗∗^	0.006^n.s.^
Use	71%	0% (0/6)	96% (24/25)	25% (1/4)	0.45^∗∗∗^	0.034^n.s.^
**VERBS_diff_**						
Exposure	66%	33% (2/6)	87% (20/23)	17% (1/6)	0.56^∗∗∗^	0.05^n.s.^
Use	80%	83% (5/6)	83% (19/23)	67% (4/6)	0.70^∗∗∗^	0.07^n.s.^
**NOUNS_diff_**						
Exposure	77%	0% (0/3)	100% (27/27)	0% (0/5)	0.15^n.s.^	0.06^n.s.^
Use	77%	0% (0/3)	100% (27/27)	0% (0/5)	0.33^∗∗∗^	0.06^n.s.^
**VOCAB_diff_**						
Exposure	74%	13% (1/8)	92% (25/26)	0% (0/1)	0.25^∗^	0.15^∗^
Use	74%	0% (0/8)	100% (26/26)	0% (0/1)	0.24^∗^	0.21^∗^


*^∗^*p* < 0.05, ^∗∗^*p* < 0.01, ^∗∗∗^*p* < 0.001.*

For MLU_diff_, language exposure was a good predictor for group membership; the model with language exposure as a predictor against a constant-only model was statistically significant (χ^2^ = 17.55, *p* < 0.001, *df* = 2), as was the model with language use as a predictor (χ^2^ = 19.64, *p* < 0.001, *df* = 2). Prediction success overall was high for language exposure and fair for language use. In both cases, the model was better at predicting group membership for the Dutch-dominant children than the balanced children and prediction success was poor for the single English-dominant child.

For UB5_diff_, language exposure and language use were equally good predictors for group membership and both models with the two predictors against a constant-only model were statistically significant (exposure: χ^2^ = 12.3, *p* = 0.002, *df* = 2; use: χ^2^ = 15.5, *p* < 0.001, *df* = 2). The model was better at predicting group membership for the balanced children than for the other two groups for both language exposure and use.

For VERBS_diff_, language use was a better predictor than language exposure for groups membership although both predictors had good prediction success (exposure: χ^2^ = 21.6, *p* < 0.001, *df* = 2; use: χ^2^ = 30.3, *p* < 0.001, *df* = 2). The model with language exposure was better at classifying the balanced children and poor at classifying the other two groups, whereas the model with language use was good at classifying both balanced and Dutch-dominant children.

For NOUNS_diff_, language exposure was not a good predictor for group membership and the model with exposure as a predictor against a constant-only model was not statistically significant (χ^2^ = 1.5, *p* = 0.22, *df* = 1). Overall prediction success was fair. However, the model could only accurately classify balanced children. The results were identical for language use (χ^2^ = 4.45, *p* < 0.05, *df* = 1).

For VOCAB_diff_, the models with language exposure and language use as predictors differed marginally from the constant only model and their overall prediction accuracy was rather poor (exposure: χ^2^ = 7, *p* = 0.03, *df* = 2, Nagelkerke = 0.25; use: χ^2^ = 6.8, *p* = 0.034, *df* = 2, Nagelkerke = 0.24). Both models had excellent accuracy at predicting groups membership for the Dutch-dominant group, but otherwise, their prediction accuracy was poor for the other two groups.

In each of these analyses, the number of cases per level of the dependent measure (i.e., the number of children per group) varied considerably and in some cases (e.g., English-dominant children in the analysis for MLU_diff_), the number of cases was extremely low. This means that the analysis may be biased and there may be complete separation in the data (King and Zeng, 2001). To address this potential problem, we re-ran the analysis using penalized maximum likelihood estimation (Firth, 1993). We used the logistf package (Heinze et al., 2016) in R (R Development Core Team, 2008) to run a penalized regression analysis. The results were comparable in terms of the prediction success, with the only difference for UB5_diff_ only: with respect to language exposure, group membership was predicted correctly for 3 out 6 (50%) Dutch-dominant children, 18 out 25 (72%) balanced children and 1 out of 4 (25%) English-dominant children, and with respect to language use, the values were 0% (0/6), 96% (24/25) and 25% (1/4), respectively. However, only the models for MLU_diff_ and VOCAB_diff_ were significant. Furthermore, the amount of variance differed across analyses with (in some cases) substantially lower values for the *R*^2^ for the penalized model than for the Nagelkerke’s *R* calculated for the standard model (compare the two rightmost columns in **Table 4**).

To summarize, language exposure and use were good predictors of group membership when this was based on differentials using MLU_diff_, VERBS_diff_ and UB5_diff_, but this did not hold for differentials using NOUNS_diff_ and VOCAB_diff_. When taking into account the small sample size, the statistical models accounted for less variance, but the overall patterns observed were comparable.

### Independently Verifying Dominance Groups and Their Relation With Language Exposure and Use

To investigate whether the dominance groups derived using the standard deviations on the various performance-based measures (differential scores) were valid, we ran two different types of cluster analysis (following Cattani et al., 2014), namely a hierarchical agglomerative cluster (HAC) analysis and a *k*-means cluster analysis using Ward’s minimum variance method (Ward, 1963) and squared Euclidean distance as the similarity measure. HAC is a bottom-up method used to determine the number of clusters in the dataset without predetermining the possible number of clusters. In a *k*-means cluster analysis, the number of (potential) clusters is pre-specified by the researcher. For both methods, we used the R package NbClust package (Charrad et al., 2014) to independently determine the optimal number of clusters. This package allows the researcher to simultaneously run up to 30 different indices, including the commonly used Gap statistic and silhouette index; the optimal number of clusters reported here is the value given by the majority of indices. We compared the clusters resulting from the HAC and *k*-means analyses with our own classification and examined the extent to which these overlapped. Subsequently, we investigated the relation between the clusters resulting from the *k-*means analysis and language exposure and use, and once again compared this to our original classification.

For both analyses, children’s scores on each measure were entered into the models without any information about either group membership (i.e., Dutch- or English-dominant or balanced) or language exposure or use. Standardly, Hopkins statistic (Hopkins and Skellam, 1954) can be used as an indication of the clusterability of a dataset. Values above 0.5 are typically interpreted as evidence that the data in a given sample are not uniformly distributed; in other words, values under 0.5 suggest that the data may not be clusterable. However, as noted by Banjaree and Dave (2004), applying Hopkins statistic to small datasets is problematic, if not impossible. We report the values here for the sake of completeness but with this caveat (*H* = 0.395 for MLU_diff_, 0.415 for UB5_diff_, 0.625 for VERBS_diff_, 0.604 for NOUNS_diff_ and 0.499 for VOCAB_diff_). For three out of the five performance-based measures – VERBS_diff,_ UB5_diff,_ and VOCAB_diff_ – the number of clusters generated by the HAC analysis was the same as in our classification (i.e., three). For NOUNS_diff_, the HAC analysis generated four rather than three clusters, and for MLU_diff_, the optimal number of clusters was between three and five, depending on the index used.

To explore the composition of the various clustering options for MLU_diff_, we generated dendrograms for the clusters proposed by the HCA. A comparison of the dendrograms for the analysis for MLU_diff_ with the least (three) and most (five) clusters generated revealed two differences. First, the three-cluster classification generated a single group at the lower end with children scoring between -1.85 and 0.10, whereas the five-cluster classification created two subgroups at the same lower end, separating one child with a score of -1.85 from the rest of the group. Second, the five-cluster classification identified an extra subgroup in the middle range of the distribution, separating children with scores between 0.24 and 0.53 from children with scores between 0.82 and 1.46, whereas in the three-cluster dendrogram these two groups were collapsed into a single group. In other words, whilst by definition more fine-grained, the partitioning of the children provided by the five-cluster classification was qualitatively comparable with the broader three-cluster classification. For this reason, we decided to adopt a three-cluster grouping in the *k*-means analysis (i.e., where the number of clusters is pre-specified by the researcher) in order to maximize the comparability of this independent means of classifying children and our original classification; given our relatively small sample size, the three-cluster option would also maximize the number of children in each cluster and for this reason was also preferable.

We adopted the same approach for NOUNS_diff_ and subsequently compared the dendrogram for the bottom-up four-cluster solution with the top–down three-way classification in order to establish their comparability. The two different cluster analyses overlapped at the upper end of the distribution (the same three participants with scores between 18 and 22 were in one cluster on both analyses). The only difference was at the lower end of the distribution: whereas the three-cluster classification generated a single group with children scoring between -20 and -5, the four-cluster classification divided these participants across two subgroups separating children with scores between -20 and -14 from children with scores between -12 to -3. In other words, at the broader level the three- and four-cluster solutions divide the group in qualitatively comparable places.

**Table 5** presents the results of the *k*-means cluster analysis, where we set the number of clusters at three. The goal of this analysis was to determine whether children were grouped in a similar way as our own classification. As such, each of the three clusters is labeled as English-dominant, Dutch-dominant or balanced, depending on the distribution of the children’s scores within that cluster. In addition, in order to compare the relationship between clusters and experiential variables across the cluster analysis and our own classification, **Table 5** specifies the number of children in each cluster with more than 65% exposure to Dutch (i.e., children who are expected to be Dutch-dominant), children with less than 35% exposure to Dutch (i.e., children who are expected to be English-dominant), as well as those who fall in between (and are thus expected to be balanced); similarly, it also specifies the number of children who use Dutch at least 90% of the time (i.e., children who are expected to be Dutch-dominant), children who use Dutch no more than 10% of the time (i.e., children who are expected to be English-dominant) and those who fall in between (and are thus expected to be balanced).

**Table 5:** Number of children assigned to each cluster (*k*-means cluster analysis), their distribution across language exposure/use groups and mean scores per measure.

Measure	Cluster	*N*	Language exposure			Language use			Mean score (range)	Comparison across clusters (ANOVA)
						
			≤35%	<65%	≥65%	≤10%	<90%	≥90%		
MLU	Cluster 1 (≈ Balanced)	19	4	12	3	3	13	3	0.86 (0.24 – 1.46)	*F*(2,34) = 68, *p* < 0.001
	Cluster 2 (≈ Dutch-dominant)	15	0	5	10	0	9	10	2.08 (1.73 – 2.67)	
	Cluster 3 (≈ English-dominant)	1	1	0	0	1	0	0	1.85	
UB5	Cluster 1 (≈ Balanced)	14	2	7	5	1	10	3	-2.36 (-6.40 – 0.40)	*F*(2,34) = 53.3, *p* < 0.001
	Cluster 2 (≈ Dutch-dominant)	19	3	5	11	2	7	10	3.87 (1 – 9.60)	
	Cluster 3 (≈ English-dominant)	2	1	1	0	1	1	0	-12.36 (-14.8 – 9.8)	
VERBS	Cluster 1 (≈ Balanced)	16	1	8	7	1	10	5	4 (-6 – 11)	*F*(2,34) = 100.2, *p* < 0.001
	Cluster 2 (≈ Dutch-dominant)	6	0	4	2	0	4	2	18.17 (13 – 25)	
	Cluster 3 (≈ English-dominant)	13	5	4	4	3	6	4	-10.23 (-16 – -4)	
NOUNS	Cluster 1 (≈ Balanced)	18	2	5	11	1	11	6	3.28 (-4 – 10)	*F*(2,34) = 68.8, *p* < 0.001
	Cluster 2 (≈ Dutch-dominant)	3	0	1	2	0	0	3	19.67 (18 – 22)	
	Cluster 3 (≈ English-dominant)	14	3	7	4	3	8	3	-11.29 (-20 – -5)	
VOCAB	Cluster 1 (≈ Balanced)	15	1	8	6	1	6	8	16.15 (3 – 15)	*F*(2,34) = 100.5, *p* < 0.001
	Cluster 2(≈ Dutch-dominant)	6	0	0	6	0	0	6	-11.13 (19 – 31)	
	Cluster 2(≈ English-dominant)	12	5	6	1	3	9	0	-11.13 (-33 – -3)	


For all measures except VOCAB_diff_, children are distributed over the different language exposure/use groups more or less as expected, with most of the children in the “balanced” cluster falling in the mid-range (<65% exposure to Dutch, <90% use of Dutch), most in the “Dutch-dominant” cluster in the highest range (≥65% exposure, ≥90% use) and most in the “English-dominant” cluster in the lowest range (≤35% exposure, ≤10% use). It should be noted, however, that there are almost always exceptions, and the relative distribution of children across the various clusters for NOUNS_diff_ and for the “English-dominant” cluster for VERBS_diff_ and VOCAB_diff_ is different; in the latter case, the “cut-off” point for dominance in English appears to lie around the 30% mark rather than 10% (cf. **Figure 2**).

As a last step, we visually examined the relationship between language exposure (**Figure 1**) and language use (**Figure 2**) with our two types of classification, that is, the original SD-based classification referred to in the Figures as *Dominance group* and represented using colors, and the *k*-means cluster analysis, represented with numbers (cf. **Table 5**).

**Figures 1**, **2** reveal that these two ways of classifying children overlap considerably. In the case of MLU_diff_, the children classified as Dutch- or English-dominant on our analysis were also grouped together in the *k-*means cluster analysis and the same holds for all but two of the 21 balanced children. A similar pattern holds for VERBS_diff_ and NOUNS_diff_, with all Dutch- or English-dominant children together in the same groups on the *k*-means cluster analysis as in our classification, and the same for 16 of the 23 balanced children for VERBS_diff_ and 18 of the 27 balanced children for NOUNS_diff_. For VOCAB_diff_ and UB5_diff_ the two means of grouping children differ slightly more: for VOCAB_diff,_ 4 of the 7 Dutch dominant children, neither the two English-dominant children and 15 of the balanced children on the SD-based classification are grouped together in the *k*-means cluster analysis. For UB5_diff_ there is overlap for almost half the children the SD and the *k*-means cluster classification. In short, then, the main difference between the *k-*means cluster analysis and our classification is that the cluster analysis grouped fewer children together in the middle of the distribution; in most cases (VERBS_diff_, NOUNS_diff_ and VOCAB_diff_), the cluster analysis grouped the children at the lower end together with the children classified as English-dominant (i.e., in red) in the original analysis.

To summarize, the hierarchical and the *k*-means cluster analyses grouped the children into largely comparable groups as the performance-based classification, and by and large the relationship between these groups, on the one hand, and language exposure and use, on the other, were also similar.

## Discussion

In this paper we examined the relationship between experiential-based and performance-based measures of language dominance in bilingual English-Dutch children in the United Kingdom and the Netherlands. More specifically, using parental questionnaire data we derived estimates of children’s patterns of language exposure and use and related these to differential scores for five variables derived from spontaneous speech data, namely morphosyntactic complexity measured by the MLU and the mean length of the longest five utterances (UB5), and lexical diversity measured by the number of different verb types (VERBS), the number of different noun types (NOUNS) and scores on a standardized vocabulary task.

### The Relationship Between Relative Exposure and Use and Relative Proficiency

Our first research question asked to what extent experiential-based factors were related to bilingual children’s relative language proficiency. The findings revealed a moderate to strong relationship between relative exposure and relative use, on the one hand, and relative proficiency as measured by MLU_diff_, VERBS_diff_, UB5_diff_, NOUNS_diff_ and VOCAB_diff_, on the other. The observation that such a relationship exists for all five outcome variables is in contrast to the original study (Unsworth, 2016a), where only MLU_diff_ and VERBS_diff_ were found to have a significant relation with language exposure and use. This difference is most likely the result of a larger sample and/or including children from across the dominance continuum. For example, the number of children with more exposure to English than Dutch more than doubled from six in the original sample to fifteen with the inclusion of the United Kingdom-resident children.

Curve-fitting analyses revealed that, as predicted, the relationship between relative experience and relative proficiency was generally best accounted for with a non-linear model. This is in line with previous research exploring differential effects of exposure and use on absolute measures of proficiency (e.g., Thordardottir, 2011; Cattani et al., 2014) and with Bedore et al. (2012) larger-scale study. It should be noted, however, that whilst more variance was captured by the non-linear models, the additional unique variance which they explain was limited (between 1 and 8%) and negligible (<1%) in certain cases.

#### Predicting Relative Proficiency Using Relative Exposure and Use, and Vice Versa

Our second research question asked whether language exposure and use could reliably classify bilingual children into language dominance groups. To this end, children were initially classified into Dutch-dominant, English-dominant and balanced groups using the standard deviation for the variable in question as cut-off point. For the number of different verb types, for example, this meant that children in the English-dominant group produced more than 12 different verb types in English than in Dutch; for the Dutch-dominant group, this pattern was reversed and for the balanced group the difference in number of verb types across the two languages was no more than 12.

By and large, the children in this larger sample patterned as predicted on the basis of the smaller sample in the original study (Unsworth, 2016a). On the whole, language exposure and use were best at predicting group membership when this was based on MLU_diff_, with accurate classification for around four fifths of the children. A similar pattern was observed for language use as a predictor of group membership based on VERBS_diff_. Classification was less accurate, but still greater than 50%, for language exposure and VERBS_diff_ and for both experiential variables as predictors of group membership based on UB5_diff_, whereas for NOUNS_diff_ and VOCAB_diff_, classification was poor. The amount of variance accounted for and the significance of the model depended on the analysis, with the clearest results for MLU_diff_ and VOCAB_diff_.

When derived independently, rather than using the somewhat arbitrary standard deviation as cut-off point, a three-cluster solution was the optimal analysis (or one of the optimal analyses) for four of the five measures (i.e., MLU_diff_, VERBS_diff_, UB5_diff,_ and VOCAB_diff_); for MLU_diff_ four- and five-cluster solutions were also considered optimal and for NOUNS_diff_ there were four clusters. In order to maximize comparability with our own classification and because the analyses with more than three clusters were at a broader level qualitatively parallel with a three-way grouping, the *k-*means cluster analysis was pre-specified at three groups. For each of these three groups, the children’s patterns of language exposure and use largely corresponded with the patterns observed in our first analysis. For example, most of the children who fell in the Dutch-dominant cluster had more than 65% exposure to Dutch and used Dutch for at least 90% of the time, at least for MLU_diff_, VOCAB_diff_, and NOUNS_diff_. In short, then, the results of the present study suggest that when relative proficiency is operationalised in terms of differentials, relative language exposure and language use can be used to classify children into dominance groups with a reasonable degree of success, and this especially holds for morphosyntactic proficiency.

### Language Exposure vs. Language Use

Children’s relative proficiency scores were related to two aspects of their language experience, namely language exposure and language use. In general, the curve-fitting analyses revealed a stronger relation between relative language proficiency and relative language use than between relative language proficiency and relative language exposure. The point at which (the majority of) children were classified as Dutch-dominant, as opposed to balanced, also differed for language exposure (around 65%) and language use (around 90%): children who were classified as Dutch-dominant used more Dutch than they were exposed to and the same pattern held for the English-dominant children, too. Note, however, that for English-dominant children, the proportion of their language use in that language was less (around the 70% mark) than the equivalent value for Dutch-dominant children in Dutch (around 90%). In short, then, children who were classified as dominant in one of their two languages were those who used one of their two languages more than they heard it.

These findings align well with a recent study on similar-aged Spanish–English bilingual children by Ribot et al. (2017). These authors found that language use at 30 months predicted rate of acquisition in English for expressive skills, as measured by a single-word picture-naming task, but not for receptive skills, as measured by a more comprehensive language proficiency task targeting various aspects of semantics, morphology, syntax and preliteracy skills. More specifically, children whose use of English at 30 months was greater than their exposure to English had better picture-naming skills in English than children for whom exposure was greater than use. Notwithstanding the fact that the tasks used to assess abilities in the two modalities were not entirely comparable, Ribot et al. (2017, p. 8) speculated that this finding might be explained in two different ways: it may reflect a more general effect of language use which was observed only for expressive skills because these are harder to achieve, or it might constitute a specific effect whereby language use specifically benefits expressive skills. In the present study, relative language use was more weakly associated with receptive than expressive skills; for some of the analyses, however, a significant relationship was observed nevertheless. In principle then, the two explanations put forward by Ribot et al. (2017) could apply here, too. The most important parallel between these two studies is the following: what seems to be crucial is not the amount of language use *per se*, but the discrepancy between language use and language exposure, that is, children who were found to be dominant in one of their two languages tended to use that language more than they heard it.

In the interests of transparency, it is worth noting that the way in which language exposure and use were calculated in the present study was not completely equivalent: the language use variable is based on the child’s language use within the home only, whereas the language exposure variable also includes sources outside the home. This means that the children resident in the Netherlands likely used (even) more Dutch than estimated here and the children resident in the United Kingdom probably used less Dutch. Incorporating these differences into our analysis would mean that the United Kingdom-resident children should shift leftward in **Figure 2**, whereas the children resident in the Netherlands would shift rightward. If anything, this would only serve to make the cut-off point between balanced and Dutch-dominant children for language use more extreme.

### Measures of Morphosyntactic vs. Lexical Proficiency

The present study applied a number of performance-based measures to spontaneous speech samples. Following previous research on language dominance in early child bilinguals (e.g., Cantone et al., 2008), morphosyntactic complexity was assessed using MLU and upper bound and children’s lexical diversity was measured using number of different noun and verb types and scores on a standardized receptive vocabulary test. On the whole, the relation between experience-based measures and performance-based measures of language dominance was clearest for MLU at the level of morphosyntax, and for number of different verb types at the lexical level. It is possible that VERBS may in part reflect morphosyntactic complexity in the sense that producing a range of verb types may in part reflect more complex, multi-verb utterances (see Unsworth, 2005, Chapter 4 for relevant discussion) and in this sense, be indicative of more complex grammatical structure. The use of nouns may be less reliable as an indicator of children’s expressive skills: children may use various referring expressions – pronouns and demonstratives or null arguments – in some contexts to represent the subject and object arguments of their verbal utterances, but none of these are included in the noun count. They may produce morpho-syntactically complex sentences, and at the same time have comparatively lower scores on lexical diversity as estimated by noun type. This may underestimate their lexical abilities and potentially lead to less variation between children for this variable. It is then perhaps unsurprising that the dominance groups based on the number of different noun types were less differentiated and bore limited (if any) relation with experiential factors.

### Coarse vs. More Fine-Grained Measures of Language Dominance

The present study highlights important differences between coarser and more fine-grained measures of language exposure. For some children there appears to be a general effect of the language of the environment, that is, there were children resident in the United Kingdom who on the basis of relative exposure (parental questionnaire data) would be expected to fall within the balanced group but who were in fact stronger in English than Dutch (cf. **Figure 1**). Similarly, the sample included children resident in the Netherlands who on the basis of relative exposure estimates were expected to fall within the balanced group but who were in fact stronger in Dutch than English (cf. **Figure 1**). It is likely that this reflects a more general effect of the language environment not captured by even the most detailed language background questionnaire (Pearson, 2007). Conversely, there were children in the Netherlands who were not Dutch-dominant and likewise, there were children resident in the United Kingdom who were not English-dominant. This was most likely because almost half of the families in the United Kingdom sample adopted a *minority language at home* approach, but there were also United Kingdom-resident children in *one parent, one language* families who were not English-dominant.

Taken together, these findings suggest that even though it is often used as such (e.g., Argyri and Sorace, 2007; Foroodi-Nejad and Paradis, 2009; Serratrice et al., 2009), language of the environment is not an accurate proxy for language dominance. This observation is consonant with recent findings by Hervé et al. (2016) and Schmeißer et al. (2016); in this latter study, the authors showed that language exposure at the individual level was a better predictor of the magnitude of cross-linguistic influence in bilingual children’s language production than language exposure at the group level (i.e., country of residence).

### Limitations and Future Research

There are a number of limitations to the present study. First, the sample size remains relatively small and the family language constellations in the United Kingdom-resident children is more varied than in the Netherlands-based children. Second, some of the performance-based measures used here, in particular MLU, may not be amenable to cross-linguistic comparison for certain languages and/or language pairs (Yip and Matthews, 2006; Allen and Dench, 2015). Third, the present study focuses on differentials as a measure of language dominance. An alternative approach would be to calculate the between-languages ratio, that is, dividing a child’s score for one language with his or her score for the other language (e.g., Sheng et al., 2014; Goriot et al., 2018) or to combine the two (Birdsong, 2016). Finally, with perhaps the exception of MLU, the values used to divide children into dominance groups are in a certain sense arbitrary in that they are sample-specific. To further assess the validity of these values for other samples, as well as the generalisability of the approach put forward here as a whole, future research should investigate different language combinations for different age groups and with different outcome measures.

## Conclusion

By using language proficiency measures commonly adopted in much of the previous literature on dominance in bilingual first language acquisition (e.g., Cantone et al., 2008) and relating these to experiential measures frequently used in the burgeoning literature on input effects, the present study brings together these two different strands of research in the field. In doing so, it expands Bedore et al. (2012) findings to younger bilingual children (i.e., 2- to 4-year-old children cf. 5 year olds) and to a different language combination (i.e., English–Dutch instead of English–Spanish). Furthermore, this study shows that relative amount of exposure and relative amount of use can be used as a proxy for language dominance, understood in its narrow sense of relative language proficiency. Crucially, however, the relation between relative language proficiency and language experience differs for these two variables. It is exactly this difference which may make it possible to distinguish between dominant and balanced children. Given that measures such as language exposure and use more readily allow for cross-study comparisons than measures which are specific to certain age ranges, languages or studies (Grosjean, 1998), and whether a child produces more than she hears is in principle relatively easy to establish, this is a welcome finding.

## Ethics Statement

This studywas carried out in accordance with the recommendations of PPLS Research Ethics Guidelines, PPLS Ethics Committee. The protocol was approved by the PPLS Research Ethics Committee, University of Edinburgh. We obtained written informed consent from all parents/guardians of the children who participated in the study in accordance with the Declaration of Helsinki.

## Author Contributions

All authorslisted have made a substantial, direct and intellectual contribution to the work, and approved it for publication.

## Conflict of Interest Statement

The authors declare that the research was conducted in the absence of any commercial or financial relationships that could be construed as a potential conflict of interest. The reviewer IF and handling Editor declared their shared affiliation.
